# Diabetic foot problem in Nepal

**DOI:** 10.3389/fendo.2023.1277940

**Published:** 2023-11-07

**Authors:** Suman Baral, Satyan Rajbhandari

**Affiliations:** ^1^ Department of Diabetes & Endocrinology Mediciti Hospital, Lalitpur, Nepal; ^2^ Department of Diabetes & Endocrinology Lancashire Teaching Hospital, Chorley, United Kingdom

**Keywords:** diabetic foot, Nepal, ulcer, neuropathy, amputation

## Abstract

**Introduction:**

Nepal is a developing country where diabetes is becoming a major health challenge due to its high prevalence of 8.5% affecting around 2 million people. Due to limited resources, there are many barriers to providing affordable and convenient diabetes care or regular screening for complications. There is no reliable data on incidence, prevalence, and complications of diabetic foot problems in Nepal.

**Methods:**

We conducted an online survey amongst senior physicians, who were members of ‘Diabetes & Endocrine Association of Nepal’ to assess their perception of diabetic foot problems in Nepal.

**Results:**

Thirty-Eight physicians responded to the survey who saw a total of 17597 patients in the preceding month. They recalled seeing 647 with 'Diabetic Foot Ulcers', giving a crude Diabetic Foot Ulcer prevalence rate of 3.7%. They recalled seeing 2522 patients with painful neuropathy that required medical treatment, giving a crude painful neuropathy prevalence rate of 14.3%. A history of foot ulcer was present in an additional 578 patients. Previous minor amputation had been performed in 215 patients (1.2%) and major amputation in 135 patients (0.8%).

**Discussion:**

Despite having expertise in various fields there is no dedicated multi-disciplinary diabetic foot clinic in Nepal. This survey shows that diabetic foot problems are abundant in Nepal and there is a need for structured multi-disciplinary approach for screening and treatment.

## Introduction

Nepal is a landlocked country in South Asia, which lies along the southern slopes of the Himalayan Mountain ranges between India and the Tibet Autonomous Region of China. It has a federal system of government with seven provinces, and the capital city is Kathmandu. The health service is the responsibility of the federal government, but some tertiary care is directly funded by the central government. There is also significant private sector involvement in healthcare in urban areas. The most recent census shows the population of Nepal to be 29 million, and the urban population has reached 66.08 percent (1). It is one of the poorest countries, with a per capita income of US$1208 (2). Although Nepal’s 2015 constitution guarantees basic health care as a fundamental right, access to high-quality care remains a privilege. The central health programs supported by international donors have reduced the prevalence of some infectious diseases, improved maternal and child health, and extended life expectancy. However, the burden of poor health in Nepal has been compounded by the rising burden of non-communicable diseases (3).

Diabetes Mellitus (DM) is one of the common non-communicable diseases that Nepal is facing as a major challenge. The International Diabetes Federation reports the national prevalence of DM amongst people aged 20–79 years old in Nepal to be 4% in 2017, which is expected to rise to 6.1% by 2045 (4). A recent population-based study sampled 13 200 participants aged 20 years and above in 400 clusters of 72 districts of Nepal. They showed the prevalence of DM to be 8.5% (5)., which is consistent with recent meta-analysis of 15 papers (6).

Although the prevalence is high, there are many barriers that people with DM commonly face in Nepal. These include accessing affordable and convenient care, receiving comprehensive diabetes education, and managing their lifestyle changes. Sub-optimal knowledge and behaviors of patients often contributed to poor DM management. The scarcity of financial and human resources for healthcare in Nepal often results in the inability of the current healthcare system to provide comprehensive management of chronic diseases (7).

Diabetic foot ulcer (DFU) is one of the chronic complications of DM. This occurs due to a combination of risk factors - namely peripheral neuropathy, peripheral vascular disease, and foot deformity. The lifetime incidence of DFU amongst diabetics is between 19-34%. The risk of death at 5 years for a patient with a DFU is 2.5 times as high as the risk for a patient with diabetes who does not have a DFU (8). There is also a high risk of amputation in patients with DFU. In Nepal, there is no reliable data on incidence, prevalence, and complications of diabetic foot problems. In a community-based survey of 34 diabetic patients in an urban area of Kathmandu, 10.5% had a foot problem in the past and 56.7% had current risk factors for DFU (9). Another study was undertaken in a tertiary care center and consisted of 169 patients with peripheral artery disease and DM. It showed that DFU was present in 32.5% of these patients (10). An outpatient clinic study from Eastern Nepal showed that 1% of the patients with DM had DFU, and none of these patients suffered from a diagnosis of peripheral vascular disease (11). Another study looked at 178 patients with diabetes mellitus. These patients were either attending out-patient appointments or were in-patient at a tertiary care hospital. The prevalence of peripheral neuropathy in this population was 41% using Michigan Neuropathy Screening Instrument (12). A study consisting of 196 patients was undertaken in a tertiary care hospital in Kathmandu. About half of these patients did not have good knowledge or practices about foot care. This data, although limited, indicates higher risk of foot complications in Nepal (13). There is no reliable data on diabetic foot problems in Nepal, and there is no information about any Multi-Disciplinary Foot Clinics in Nepal.

## Aim of the study

The aim of this study was to use a questionnaire directed at Diabetes Physicians in Nepal to explore the resources available for the management of diabetic foot problems, and to explore the extent of any problems regarding this management.

## Methods

This was a semi-quantitative survey amongst senior physicians, who provide diabetes treatment and were members of Diabetes & Endocrine Association of Nepal (DEAN). It assessed their perception of diabetic foot problems in Nepal. All Viber group members of DEAN were invited to take part in a short online survey (**Table 1**) in May 2023 about their experience of treating patients with diabetes in the last one month.

**Table 1:** List of online questions sent to ‘Viber group’ members of Diabetes & Endocrine Association of Nepal (DEAN).

• **What is your name?**
• **Which city/village do you practice?**
• **On average how many patients with diabetes do you see every month?**
• **Of those patients with diabetes seen every month how many have ‘Diabetic Foot Ulcers’ (Ulcers below ankle)?**
• **Of those patients with diabetes seen every month how many have painful neuropathy that needs medical treatment?**
• **Of those patients with diabetes seen every month how many have had at least one diabetic foot ulcers in the past?**
• **Of those patients with diabetes seen every month how many have had toe amputation (minor amputation) in the past?**
• **Of those patients with diabetes seen every month how many have had amputation of leg (Major amputation) in the past?**
• **Do you have a good system of annual foot screening in your practice?**
• **Do you have good access to Podiatrists in your multi-disciplinary team?**
• **Do you have good access to radiology (For X-ray and CT/MRI) from your clinic if needed?**
• **Do you have good access to vascular surgeons from your clinic?**
• **Do you have good access to interventional radiologists (for peripheral angiogram and angioplasty) from your clinic?**
• **Do you have good access to Orthopaedic surgeons from your clinic?**
• **Do you have good access to Orthotist (custom made shoe maker) from your clinic?**
• **Do you have good access to microbiology service from your clinic?**
• **Do you have good access to off loading (eg plaster technician) from your clinic?**
• **How confident are you in managing ‘Diabetic Foot Problem’**
• **Are you interested in further training to manage diabetic foot problems?**
• **Any comments?**

### Inclusion criteria

- Doctors actively treating Diabetes in Nepal
- Members of DEAN
- Contributes to Viber Group of DEAN

### Exclusion criteria

- Doctors not using mobile phone
- Doctors who did not respond to reminder

The questionnaire was kept short and user friendly, which could be completed on a smartphone. The questions were based on those used in data collection for National Diabetes Audit in the UK. After two weeks, reminders were sent to physicians who did not respond to the initial invitation. If the results were given in a range, the middle figure was chosen and if required was rounded to the nearest whole number. Simple descriptive analysis was done using Excel.

## Results

Out of 96 eligible physicians, 38 (39.6%) completed the survey. All physicians were practicing in urban centers, with 21 (55.3%) practicing in the capital and 28 within the greater Kathmandu Valley, which consist of capital city of Kathmandu along with Patan and Bhaktapur cities. All these physicians saw a total of 17597 patients in the preceding month. They recalled seeing 647 with ‘Diabetic Foot Ulcers’ (Defined by ulcers below the ankle), giving a crude Diabetic Foot Ulcer prevalence rate of 3.7%. They recalled seeing 2522 patients with painful neuropathy that required medical treatment, giving a crude painful neuropathy prevalence rate of 14.3%. A history of foot ulcer was present in an additional 578 (3.3%) patients. Previous minor amputation had been performed in 215 patients (1.2%) and major amputation in 135 patients (0.8%) (**Table 2**).

**Table 2:** Presence of diabetic foot pathology amongst 17597 patients.

Condition	Number	Percentage
Active Diabetic foot Ulcer	647	3.7%
Active Painful Neuropathy	2522	14.3%
Previous foot Ulcer (Now healed)	578	3.3%
Previous Minor Amputations	215	1.2%
Previous Major Amputations	135	0.8%

Regarding the presence of a multi-disciplinary team, Diabetes Physicians had access to podiatrists in only 5 (13.2%) centers; with the 33 centers (86.8%) not having access to foot care practitioners. In contrast, all physicians had good access to orthopedic surgeons and microbiology services. All modalities of investigations including MRI were available to 35 (92.1%) physicians, with one having access to X-Ray only and two having access to none. 30 (78.9%) physicians had access to vascular surgeons within their center, and 23 (60.5%) had good access to interventional radiologists (for peripheral angiogram and angioplasty). Plaster technicians were available in 18 (47.4%) centers, but we did not collect data on whether they were actively used in clinics to manage diabetic foot ulcers. Only nine clinics (23.7%) had good access to orthotists, with the majority (76.3%) not having any facilities for custom made shoes (**Table 3**).

**Table 3:** Available facilities for multi-disciplinary management of diabetic foot ulcer in 38 centres.

Facility	Number	Percentage
Podiatrist on site	5	13.2%
Orthopaedic surgeons	38	100%
Vascular surgeons	30	78.9%
MRI	35	92.1%
No radiology facility	2	5.3%
Interventional Radiologist	23	60.5%
Plaster Technician	18	47.4%
Orthotist	9	23.7%

Of all practicing physicians, 25 (65.8%) had a good system of annual foot screening. When broken down to the number of patients these physicians see, 12090 (68.7%) patients had annual diabetic foot screening. The exact details of such screening program were not collected. When asked to score numerically their confidence in managing ‘Diabetic Foot Problems’ in with 10 being most confident, physicians gave an average score of 6.7. Thirty-five (92.1%) physicians were interested in receiving further training to manage diabetic foot problems.

## Discussion

This is the first study that has collected data from physicians treating diabetes on their experience of the management of foot problems in Nepal. There is no clear distinction between primary care, secondary care, and tertiary care in Nepal, so the sample collected was across all these levels. All physicians were practicing in urban areas, so there is no data on rural populations. However, according to the most recent census, 66% of the Nepalese population live in urban area (1). Urban residents had almost double the risk of developing diabetes when compared with rural residents (5), so our data is likely to be representative of the national problem. The prevalence of ulcers in this study (3.7%) is lower than the reported global prevalence of 6.3% (14). In a similar study from India, Diabetic foot ulcers were found in 4.54% of newly diagnosed type 2 diabetes mellitus patients (15), which is similar to our findings. Our sample did not collect data on new or follow up patients and whether they were attending primary or secondary care settings.

There is no dedicated Multidisciplinary Diabetic Foot Clinic in Nepal. The earliest Multidisciplinary Diabetic Foot Clinic was established in 1981 at King’s College Hospital in London, UK (16). After that, many Multidisciplinary Diabetic Foot Clinics were established all over the world, which has led to a reduction in amputations. One study in New Zealand showed that after the conception of a multidisciplinary diabetic foot clinic, there was a reduction in hospital admissions, a seven-fold reduction in major amputations, a reduction in mortality rate by 50%, and an overall reduction in total costs (17). Therefore, it is important that steps are taken to set up such clinics in Nepal, providing there is a suitable workforce and suitable facilities available. There is a distinct lack of podiatrists and orthotists in Nepal. As there is no podiatry training scheme in Nepal, interested nurses can be trained to specialize in foot care. Similarly, local people can be trained to manufacture suitable footwear for diabetes patients. These have been practiced in local leprosy hospitals with remarkable success (18). The initiation of a Multidisciplinary Diabetic Foot Clinic is of great importance. As the majority of diabetes physicians are interested in learning about diabetic foot problems, appropriate training programs should contain information about how to set up and refer to new Multidisciplinary Diabetic Foot Clinics (19).

Of all reported patients on our study, only 68.7% had annual foot screening. This number is expected to be much lower in rural area, where even implementing access to basic health care is difficult. In 1988, the government of Nepal introduced the Female Community Health Volunteers. They are frontline workers in the Nepalese healthcare system and are trusted by community members. There are over 51,000 members in Nepal as of the end of 2020 (20). They can be trained in annual diabetic foot screening and foot education.

The current Nepalese healthcare system, consisting of mixed public and private funding, is not equipped to manage the growing number of people with diabetes; and is unable to provide adequate prevention, diagnosis, and management services for diabetes (7). A multilevel, coordinated approach is necessary to bridge the current gap between the community and the health system to ensure equal access to diabetes services for all Nepalese people. In Nepal, the national health insurance program was first introduced in 2015. This now covers the whole country. It gives special financial relief to patients who suffer from conditions such as heart disease and chronic kidney disease requiring dialysis. It does not include financial aid diabetics, which is a key risk factor for these conditions (21). There is also a problem in the uptake and retention of patients in this scheme. There is an imbalance between the population’s expectations of health insurance and the institutions who deliver the insurance (22). Therefore, it is essential that policy makers in Nepal highlight the importance of managing chronic conditions like diabetes in their national health insurance program. There should be regulatory structures to define and ensure that a minimum quality of care is delivered; at all levels within both public and private health-care institutions.

## Strengths and limitations of this study

This was a small survey, and data was based on recollection of cases rather than independent review of case notes. In Nepal, most diabetes service in urban areas are provided within the private sector, and the record keeping is not always adequate. In general, patients tend to visit various institutions for their numerous health conditions; rather than stick to a single one provided by either the public sector or by an insurance scheme. Therefore, reviewing of records from a single institution is unlikely to be reliable. As this was a study based on physician’s perception of the problem in an average month, there could be a recall bias. A key limitation in this study was that we did not get any responses from physicians practicing in rural areas. This is mainly because private diabetes physicians practice in urban areas, and secondary care hospitals are also based in urban areas.

## Conclusions

Our survey captured approximately 40% of all physicians practicing diabetes management in Nepal. DEAN is the only organization where all such physicians become a member. The DEAN Viber group is very active in promoting communication between professionals. Therefore, we believe that we were able to capture recall data on a large number of patients. The preliminary findings from this physician survey show a lack of community foot protection with very few podiatrists, and a need for development of Multidisciplinary Diabetic Foot Clinics. In addition, the work should be followed up with a prospective audit of outcomes for persons living with diabetes who suffer diabetic foot ulceration.

## Data availability statement

The original contributions presented in the study are included in the article/supplementary material. Further inquiries can be directed to the corresponding author.

## Author contributions

SR: Conceptualization, Writing – review and editing. SB: Conceptualization, Data curation, Formal Analysis, Writing – review and editing.
